# Supplementary material for: Sex Differences in Individuals at High Risk of Atrial Fibrillation: A Primary Care Community Cohort Study, 2015–2024
Source: Biomedicines. 2025 Nov 18;13(11):2814. doi: 10.3390/biomedicines13112814 (PMC12650675; doi:10.3390/biomedicines13112814)
Supplement: Supplementary file 1 [file biomedicines-13-02814-s001.zip › biomedicines-3945185-supplementary.pdf]

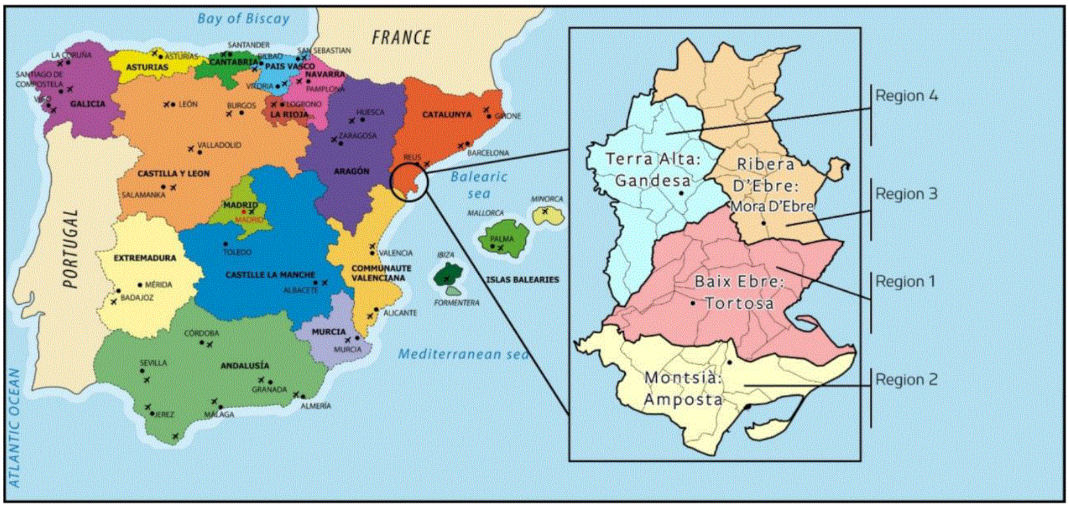

Figure S1. Spanish map and the territory *Terres de l'Ebre*.

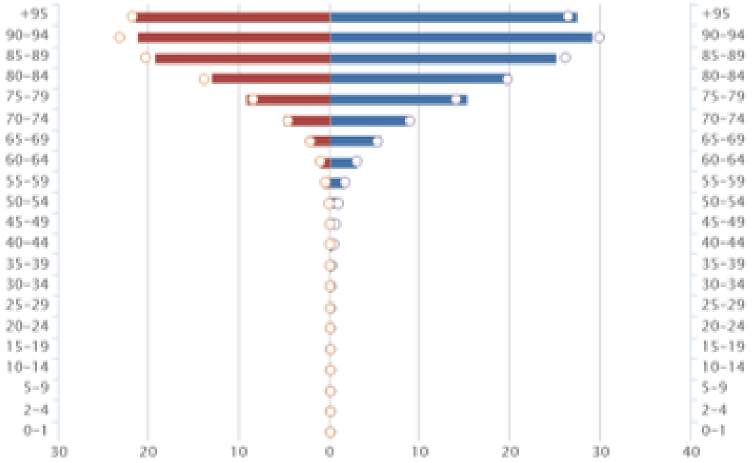

**Total**	3.24	2.61	2.93
**+95**	27.43	21.58	23.24
**90-94**	29.06	21.29	23.95
**85-89**	25.06	19.53	21.81
**80-84**	20.03	13.19	16.22
**75-79**	15.29	9.32	12.08
**70-74**	9.04	5.13	7.04
**65-69**	5.68	2.59	4.13
**60-64**	2.93	1.21	2.06
**55-59**	1.62	0.76	1.19
**50-54**	0.98	0.33	0.68
**45-49**	0.6	0.26	0.44
**40-44**	0.32	0.06	0.2
**35-39**	0.19	0.06	0.13
**30-34**	0.11	0.07	0.09
**25-29**	0.02	0	0.01
**20-24**	0.04	0.02	0.03
**15-19**	0	0.02	0.01
**10-14**	0	0	0
**5-9**	0	0	0

Figure S2 Prevalence of Atrial Fibrillation in the territory *Terres de l'Ebre* (May 2025).

Table S1. Summary of sex differences in patients at high-risk of AF (quartile 4) and new AF

Group	Women	Men
**Quartile 4 Inter-sex**	Hypertension, body mass index (BMI), cognitive impairment, Pfeiffer score, dyslipidemia, mortality.	Diabetes mellitus, stroke, peripheral vascular disease, ischemic cardiomyopathy, Charlson index, OSAHS
**[new AF] vs [Quartile 4] Intra-sex**	Heart failure, stroke, peripheral vascular disease, ischemic cardiomyopathy, Charlson index, chronic kidney disease (CKD), obstructive sleep apnea–hypopnea syndrome (OSAHS).	Heart failure, stroke, Charlson index, CKD, OSAHS, and mortality.
**new AF Inter-sex**	Age, BMI, Pfeiffer score.	Diabetes mellitus, peripheral vascular disease, ischemic cardiomyopathy, Charlson index, OSAHS.
